# Three-dimensional analysis of the heart function and effect cholinergic agonists in the cockroach *Gromphadorhina portentosa*

**DOI:** 10.1007/s00359-020-01443-5

**Published:** 2020-09-21

**Authors:** Alfonso Claros-Guzmán, Martín G. Rodríguez, Birmania Heredia-Rivera, Rodolfo González-Segovia

**Affiliations:** 1 Department of Pharmacology and Physiology, Autonomous University of Aguascalientes, 940 Av. Universidad, Aguascalientes, 20130 México; 2 Department of Microbiology, Autonomous University of Aguascalientes, 940 Av. Universidad, Aguascalientes, 20130 México

**Keywords:** Dorsal vessel, Intracardiac valves, Acetylcholine, Cardiovascular, Nicotine

## Abstract

Many relevant aspects of mammal’s cardiac physiology have been mainly investigated in insect models such as *Drosophila melanogaster* and *Periplaneta americana*. Cardiac function has been poorly studied in the cockroach *Gromphadorhina portentosa,* which has some advantages for experimental purposes such as an easier culture, bigger organs and a robust physiology. On the other hand, the study of cardiac physiology in insects has been largely improved since the arrival of digital imaging technologies for recording purposes. In the present work, we introduce a methodology of video recording coupled to an isotonic transducer for a three-dimensional analysis of the heart and intracardiac valves of *G. portentosa*. We used this methodology for assessing the physiological responses of the cockroach heart upon the application of different cholinergic neurotransmitters (acetylcholine, nicotine and muscarine). We recorded in detail the relationship between intracardiac valves movement, hemolymph flow, diastole and systole. Acetylcholine and nicotine induced a biphasic effect on the cardiac frequency. Acetylcholine increased the diastolic opening. Nicotine at high concentration caused paralysis. Muscarine induced no major effects. These findings suggest a combined action of cholinergic agonists for a finely tuned the cardiac frequency, intracardiac valves function and cardiac cycle.

**Electronic supplementary material:**

The online version of this article (10.1007/s00359-020-01443-5) contains supplementary material, which is available to authorized users.

## Introduction

Although mammals and insects are phylogenetically very distant, their physiology is unexpectedly very similar. For example, the heart activity assessment by current electronic recording techniques have demonstrated that pulsations of insect and human hearts are regulated by similar myogenic mechanisms based on the depolarisation of myocardial cells (Sláma 2012). Therefore, the study of the cardiovascular system in insect models remains relevant biological research. These similarities between insects and vertebrates extend to basic elements of development, functions and aging (Berh et al. 2018), for instance various studies indicate a number of genes in *D. melanogaster* implicated in cardiac function regulation, muscle contractile proteins and ion channels that are similar to those in mammals (Akasaka et al. 2006; Malloy et al. 2016).

The main propulsion organ in insect circulatory system is the dorsal vessel this cavity is located underneath the dorsal epidermis and circulates insect hemolymph between the three major body compartments (head, thorax, and abdomen) which are mutually interconnected and form the so called “hemocoelic cavity”. Likewise to mammalian blood, the hemolymph provides nutrients, gas exchange and signalling molecules to different the body compartments. The dorsal vessel in adult insects consists of a narrow elastic tube called the thoracic aorta and a larger abdominal portion that is called heart. Its myogenic function is characterised by a very regular reversal flow with three distinctive phases: (a) a backward oriented (retrograde) cardiac pulsation; (b) a forward-oriented (anterograde) pulsation with faster frequency; and (c) shorter or longer periods of temporary cardiac standstill that usually occurred after the termination of the anterograde phase (Sláma 2003). A unidirectional flow of hemolymph is assured by the intracardiac valves by closing the luminal space and blocking the back-flow (Rotstein and Paululat 2016). In contrast to human heart, the dorsal vessel is mostly dedicated to maintaining hemolymph flow only in the hemocoelic cavity and a specialised set of accessory pulsatile organs (APOs, or auxiliary hearts) that contributes toward the circulation in minor capillaries. Cardiac function in insects also requires endogenous substances which have been characterised as common neurotransmitters present in the hemolymph and affecting many peripheral and non-neuronal tissues (Miller and Melcalf 1968; Malloy et al. 2016). Some of these neuromodulators are the biogenic amines that are known to increase the rate of heartbeat in semi-isolate heart preparations from several insects. However, the regulation of the cardiac physiology by the cholinergic system remains poorly understood (Malloy et al. 2016).

On the other hand, several methodologies have been proposed for recording the heart function (Berh et al. 2018) such as multielectrode array systems (impedance converter), multiple sensors electrocardiography (optical method or isotonic transducer) (Miller 1979; Sláma 2003; Wasserthal 2007), atomic force microscopy, optical coherence tomography (OCT) or Doppler OCT (Berh et al. 2018), Frustrated Total Internal Reflection (FTIR) or FTIR-based imaging method (FIM) (Risse et al. 2013), Synchrotron X-ray phase-contrast imaging (Lee and Socha 2009), Dye angiography technique, real-time microscopic imaging (Choma et al. 2011), live body injected with red fluorescent microspheres and muscle fluorescent labelling (Glenn et al. 2010). However, until now, there are no studies that include an analysis of the conformational changes of the heart that occur during the insect cardiac cycle (systole and diastole) observed by a three-dimensional approach. Moreover, neither differentiation nor the biomechanical functionality of valve cells has been described in detail (Rotstein and Paululat 2016).

Although comparative physiology has been more studied in drosophilids, the physiological analysis of heart function in this kind of organisms can be a challenging task because they are small and have a high pulse rate of the heart. Cockroaches remain an interesting alternative as they are larger, inexpensive, resistant to disease and trauma, insensitive to pain, and that many functional analogues can be made between cockroach and mammalian systems. Specifically, most of the reports on cardiac function have been done in *Periplaneta americana* (Krijgsman and Krijgsman-Berger 1951; Ludwig et al. 1957; Miller 1968a, b; Collins and Miller 1977; Pass et al. 1988)*.* In contrast, the hissing cockroach of Madagascar (*Gromphadorhina portentosa*) has drawn less attention, nevertheless it has some advantages for experimental physiology as it is more robust and relatively easier to culture.

In the present study, the effects of three cholinergic agonists (acetylcholine, muscarine, and nicotine) on the heart and the intracardiac valves of the cockroach *G. portentosa* (Schaum) were evaluated by combining two different physiological recording techniques: isotonic transducer and video-record analysis. This novel approach allows a three-dimensional assessment of the in vivo heart activity on a reliable biological preparation from *G. portentosa*.

## Methods

### Biological material

The cockroaches (*G. portentosa*) were maintained according to Mulder and Shufran (2017). The semi-isolated cockroach heart preparation was carried out according to Jones (1974). Only males were used; briefly, cockroaches were anesthetised using CO_2_. Head, legs and entire ventral surface were removed. The ventral midline was cut through and all the viscera were removed. Before each experiment, every semi-isolated heart preparation was briefly soaked in a temperature-controlled bath (25 °C) containing a physiological saline (200.17 mM NaCl, 10.73 mM KCl, 0.99 mM MgSO_4,_ 3.4 mM CaCl_2_, 2.14 mM NaHCO_3_, 0.083 mM NaH_2_PO_4_) adjusted to pH = 6.9 (Collins and Miller 1977). During the experiment, a constant volume of physiological saline was maintained in the dorsal vessel using a micropipette. The physiological saline caused no alteration in heart functions.

### Experimental procedures

Experimental doses of acetylcholine (ACh), nicotine and muscarine were diluted in physiological saline and perfused by applying one drop (20 μl) directly onto the heart. The applied doses were of 5.5 and 1 mM of ACh chloride (Sigma-Aldrich catalogue number: A6625); nicotine at 0.53 and 5.3 µM (Sigma-Aldrich N3876-100ML); muscarine at 0.01, 1 and 5.5 mM (Sigma-Aldrich M6532-5MG). The experimental doses of ACh and nicotine were taken from the average concentration that caused a significant effect (increased cardiac frequency, although smaller doses decreased cardiac frequency) on *Periplaneta americana* and *Drosophila melanogaster* hearts (Miller 1968b; Malloy et al. 2016). The muscarine doses were similar to those of ACh. Biological replicates consisted of 6 cockroaches for each experimental procedure; heart preparations with exclusively physiological saline were assessed for 6 min as the control period. Then, the experimental doses were added, and heart function was monitored for six minutes. All experiments were performed on the 3th abdominal segment.

### The *z*-axis approach

The cardiac cycle of cockroach consists of two mechanical movements: a systolic phase (closing) and a diastolic phase (opening). The systolic phase occurs when the heart walls contract and close the luminal space of the heart tube. When the heart walls relax the luminal space opens and the diastolic phase has taken place. These heart events have been studied by video record and movement analysis in two dimensions (Dulcis 2005; Wasserthal 2007).

**Fig. 1:**
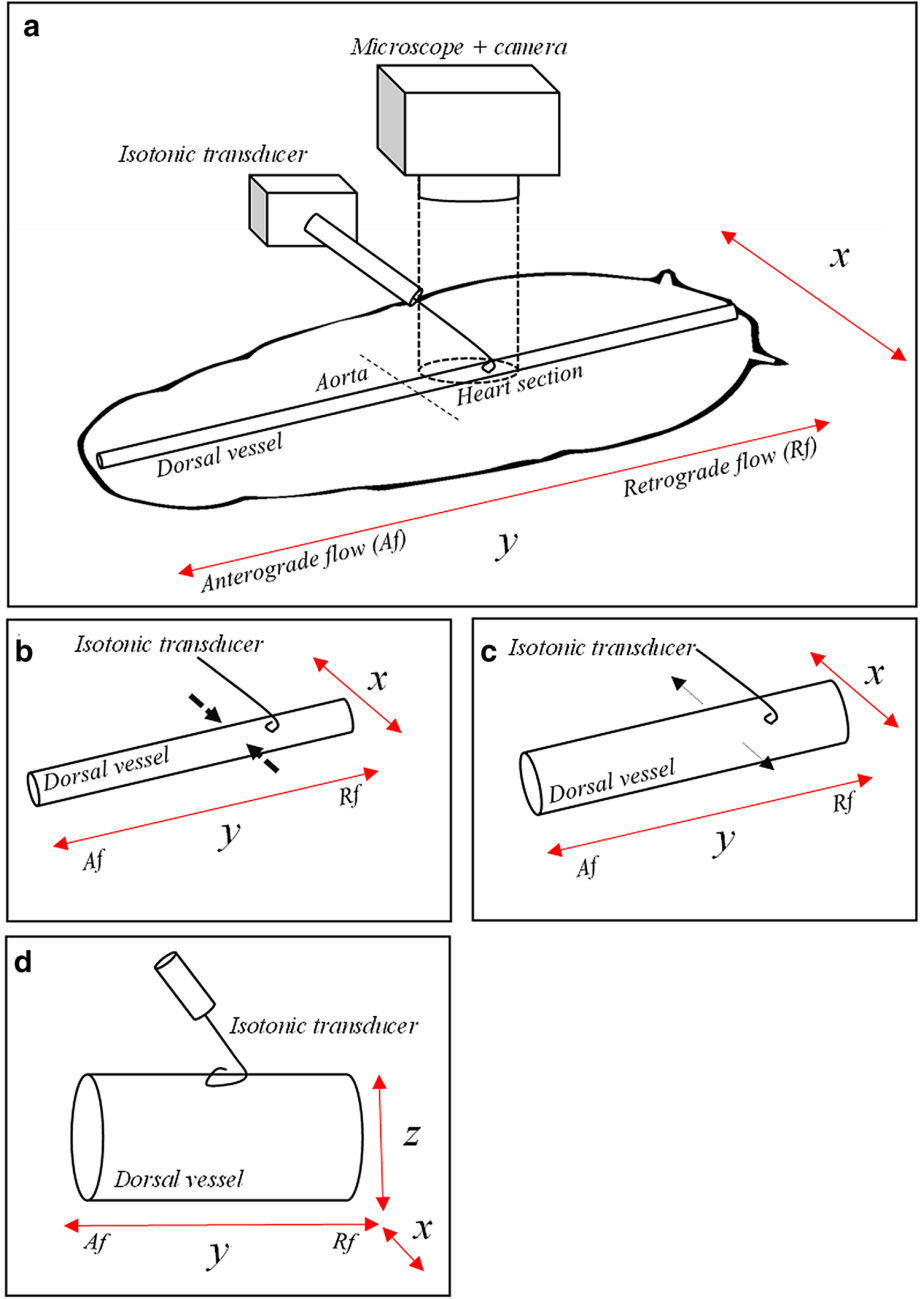
Schematic representation of a semi-isolated heart physiological preparation of *G. portentosa*. **a** Ventral side of the whole body of *G. portentosa* and heart function recording setup which consisted of the isotonic transducer and the camera coupled to the stereoscopic microscope. The dorsal vessel is divided into two sections: aorta and heart. The hemolymph flow is indicated on the *y*-axis. Both the systolic contraction (**b**) and the diastolic opening (**c**) were observed by video recording on *x-*axis. A wire tip was adapted to the isotonic transducer for registering the heart movements on *z-*axis (**d**)

Nevertheless, the mechanical movement of the heart is more complex, since it involves changes in diameter and hemolymph flow direction. We hypothesised that this phenomenon could be explored by an analysis of three dimensions. Therefore, we developed a new method by complementing video recording with a z movement assessment. The *x* and *y*-axis of the cockroach heart preparations are assessed by a camera as in Fig. 1. We used A “Harvard” type isotonic transducer (apparatus heart/smooth muscle transducer) coupled to Biopac System MP150 for registering the movements of the cardiac cycle in *z-*axis direction (dorsal and ventral movements). When the systolic contraction take place, the isotonic transducer records a decaying signal, whereas the diastolic opening is recorded as a raising signal. These movements in the *z-*axis are very smalls and expressed as millivolts by the displacement transducer. The Acknowledge 4.1 software (BIOPAC Systems, Inc.) was used for data acquisition and analysis of cardiac frequency and diastolic amplitude. The isotonic transducer data were filtered and smoothed online to avoid small signals resulting from motion artefacts. The cardiac frequency (Hz) was the frequency of predominant waves on power spectral analysis. The diastolic amplitude of the heart was considered as the mean value of movement on the z-axis. The amplitude values are reported per minute and expressed in percentage. The hemolymph flow direction was referred to as anterograde and retrograde based on the *y-*axis.

The video recordings of cardiac activity were done with a Panasonic GP-KR222 camera coupled to a stereoscopic microscope (CCSM, ZEISS Stemi DV4), some videos were captured with a digital Celestron 44,422 HD camera. The diastolic opening was quantified by selecting three frames at specific time points of each video record minute (first photo 0–15 s, second photo 30–40 and third photo 50–60 s). The photos were analysed by ImageJ software. The measurements were performed considering two points related to the diastolic opening movement.

The cardiac events assessed by video record was always synchronised to the isotonic transducer signal thanks to the BIOPAC software. The recordings of intracardiac valves were captured with the CCSM. Because the valves are not always observed at the same focal plane, some images appeared with different contrast.

### Statistical analysis

The statistical analyses were done using ANOVA and Tukey’s Honestly Significant Difference (HSD) test to determine significant differences between control and experimental conditions. Each experimental condition was tested on six cockroaches. The data were analysed using statistical software Graph Pad Prism with one-way ANOVA (*N* = 6, *p* value < 0.05).

## Results

### Assessing the relationship between intracardiac valves and heartbeats by video/transducer synchronisation

**Fig. 2:**
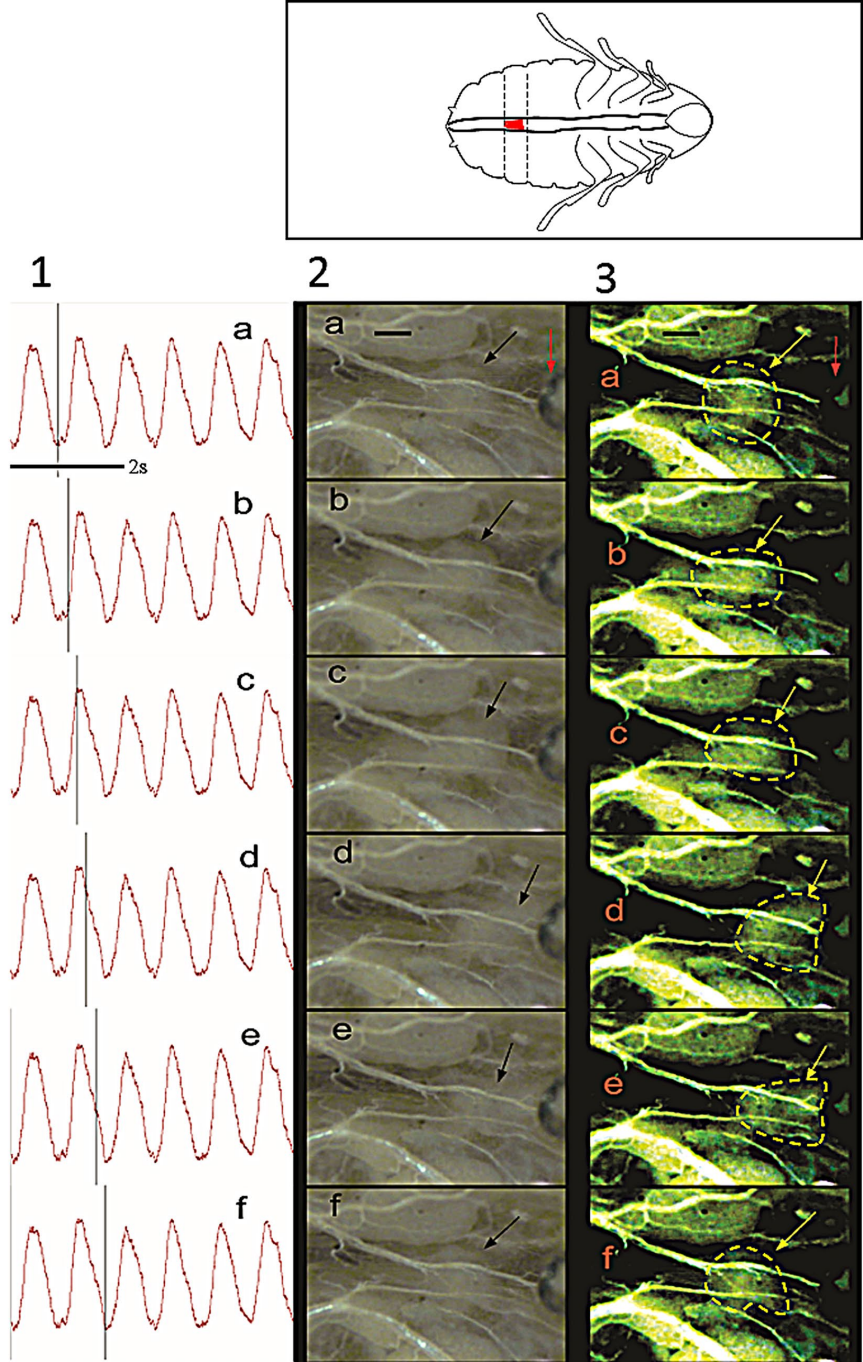
Synchronised recording of heart diastole and systole with the Biopac MP150 and video recording. The schematic cockroach (top right) shows the selected intracardiac valve (in red) located at 3th abdominal segment of the cockroach heart. The schematic cockroach also indicates the viewing (ventral side) and orientation of the video frames (anterior = right, posterior = left). The columns 1–3 show: the z movement recording by the transducer, original frames, and the same frames contrasted by ImageJ, respectively. The black line means scale (200 µm). The black and yellow arrows, and yellow contour indicate the valve. The red arrow indicates the position of the isotonic transducer sensor. The frames were taken from Online Resource 1

To validate our methodology for cardiac function recording, we evaluated the intracardiac valve contractions of semi-isolated preparations of *G. portentosa* hearts. Cardiac events were effectively characterised by video/isotonic transducer synchronisation, our observations indicated a well-established relationship between the movement of the intracardiac valves, the volume of hemolymph in the heart, the diastole and systole. The relationship between the heart and the movement of the intracardiac valves can be described in four stages that drive the hemolymph flow, the opening of the heart (diastole) and contraction of the heart (systole). These stages can be clearly identified by video recording. In the first stage (systolic stage), the valve is contracted, and it extends at posterior direction (Fig. 2a and b). The second stage (diastolic stage) begins when the valve closes the luminal space (Fig. 2c and d). The third stage consists of a valve flipping to the anterior direction and it is followed by the initiation of a new systolic stage (Fig. 2e). During systolic phases, the valve quickly moves to a posterior direction and contracts (Fig. 2 f). The closure of the intracardiac valve restricts the hemolymph flow (Fig. 2d) and the heart lumen increases until reaching a maximal volume. At this time point, the intracardiac valve reaches its closest position to the anatomical head (Fig. 2e). Additionally, cardiac events were clearly depicted by the isotonic transducer recording as each signal peak seems to correspond to the maximal volume raise in the luminal space, whereas a decreasing signal amplitude correlates to a decay in volume as shown in the video (Online resource 1).

The movement of the intracardiac valves during the second and third stages seemed to prevent hemolymph reflux. Therefore, the hemolymph flux across the entire dorsal vessel was assessed using fluorescein as a flow marker. An intact moulting cockroach floating on a fluorescein solution was recorded, the dorsal vessel was contrasted by dark light. The video analysis (Online resource 5) suggests that hemolymph displacement across the dorsal vessel could be facilitated by a peristaltic movement. This experiment also indicates that anterograde flow initiates with a big abdominal contraction, which propagates from the heart to the aorta and is repeated every ~ 1.5 ± 0.1 s (Fig S1, Online resource 5). A fluorescein drop was placed at the dissected anterior part of the dorsal (Online resource 6), the fluorescent marker filled the aorta during the diastolic movement, but it was effectively blocked by the intracardiac valve and did never enter into the heart section indicating that this set of valves help to maintain the anterograde flow during heart pulsations.

### Acetylcholine accelerates heart function and restricts the intracardiac valve movement

**Fig. 3:**
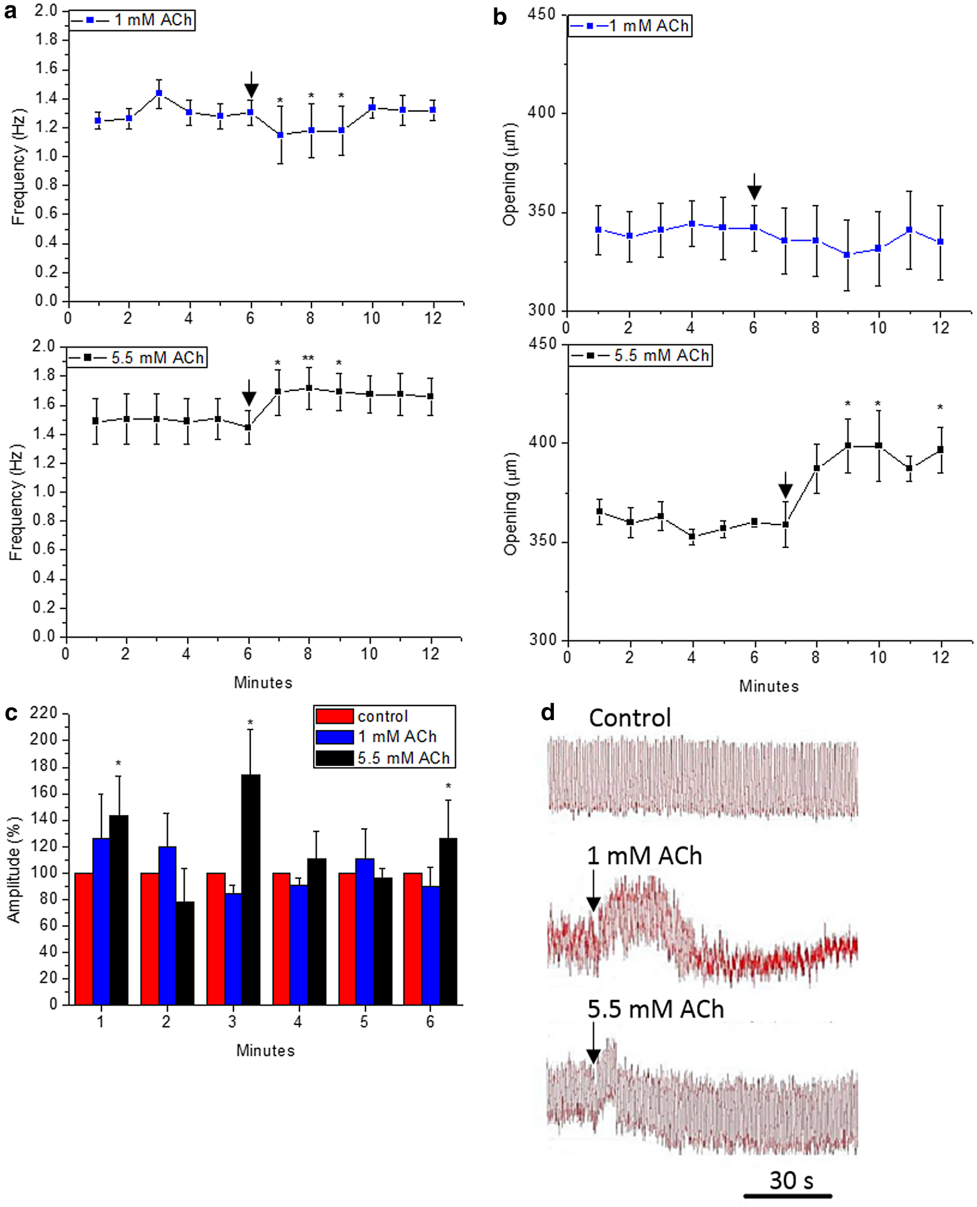
Effects of ACh (1 mM) on the heart and intracardiac valve recorded by synchronised isotonic transducer coupled to Biopac MP150 and video recording. The schematic cockroach (top right) shows the selected intracardiac valve (in red) located at 3th abdominal segment of the cockroach heart. The schematic cockroach also indicates the viewing (ventral side) and orientation of the video frames (anterior = left, posterior = right). The column 1–3 show: the isotonic transducer recording, original frames, and the same frames contrasted by ImageJ, respectively. The black line means scale (200 µm). The black and yellow arrows, and yellow contour indicate the valve. The red arrow indicates the position of the isotonic transducer sensor. The frames were taken from Online Resource 2

Cholinergic compounds, such as ACh, are commonly described as cardioaccelerators in insects (Miller and Metcalf 1968; Miller 1968b; Malloy et al. 2016). We proceeded to record and characterise the cholinergic effect of ACh in *G. portentosa*. The normal operation of the intracardiac valve was clearly disturbed by the addition of ACh at 5.5 mM. Systolic and diastolic phases registered by the isotonic transducer (Fig. 3, column 1) were irregular in amplitude and their kinetic appears more undifferentiated. The valve has a restricted movement and hemolymph displacement appears dramatically decreased (Fig. 3, column 1 and 2) compared to the control period. The video record suggests that the muscles attached to intracardiac are also affected by the high concentration of ACh.

**Fig. 4:**
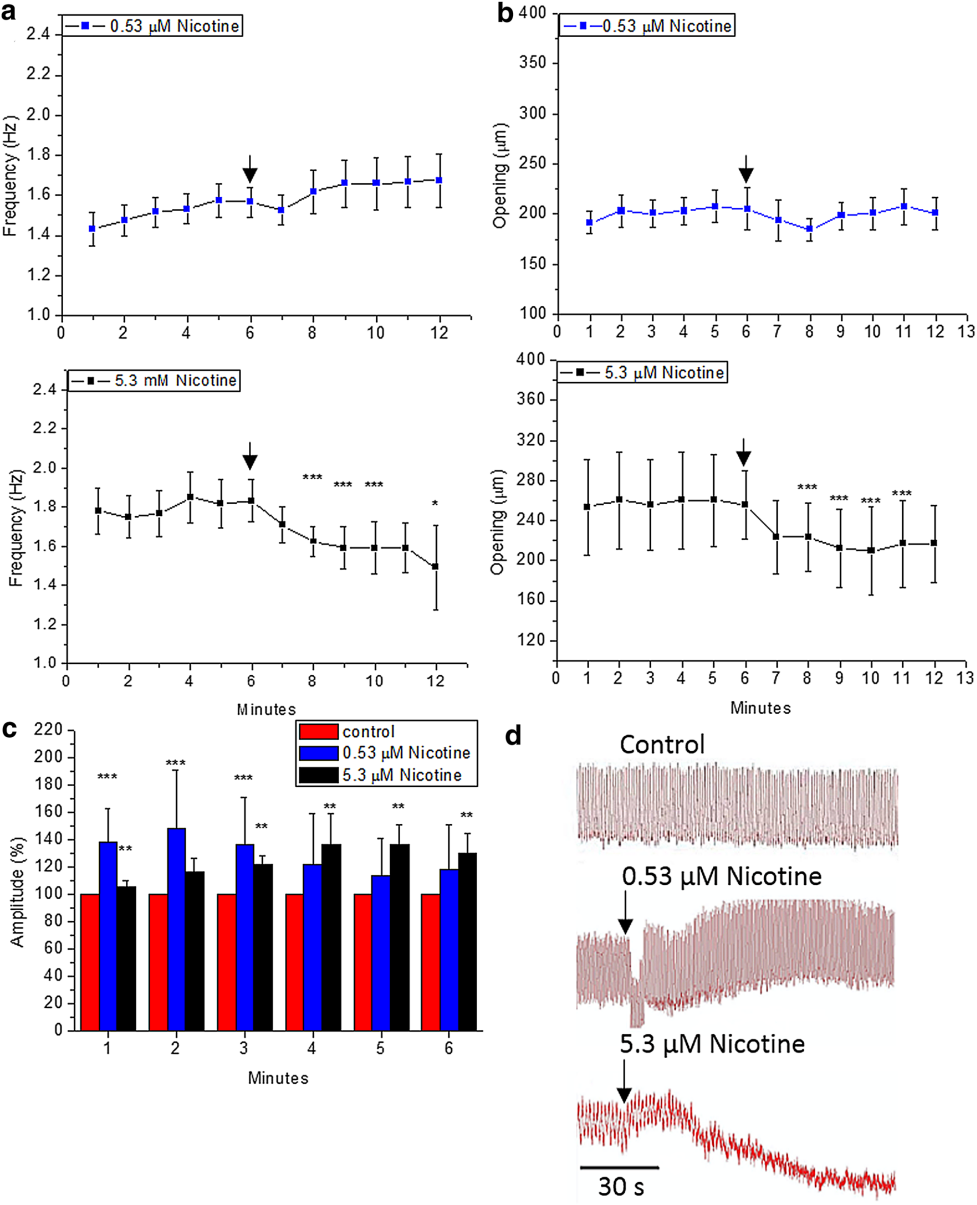
Effects of the addition of ACh (1 mM and 5.5 mM) to semi-isolated heart preparations of *G. portentosa*: **a** cardiac frequency, **b** opening of heart, and **c** the diastolic amplitude of heart. The amplitudes after ACh addition are expressed as 100% of the control amplitude. Data shown are mean values and error bars indicate SD (*n* = 6). **d** Representative traces of heart activity. The downward-facing arrows indicate the time of addition of ACh. Differences attributed by Post Hoc TSD Tukey Test. *p* value code ‘***’ < 0.001, ‘**’ < 0.01, ‘*’ < 0.05, ‘.’ < 0.1 and ‘’ = not significant

Interestingly, an ACh concentration of 1 mM induces initially a slight decrease of cardiac frequency (Fig. 4 a) which eventually recovers the basal frequency (~ 1.3 Hz). The addition of ACh at 5.5 mM increases considerably the heart frequency (~ 1.7 Hz). This higher concentration of ACh not only affected the cardiac frequency but also significantly increased the diastolic opening (Fig. 4b). In contrast, the addition of 1 mM of ACh was followed by a minor decrease of diastolic opening.

The effect of ACh was also characterised by an initial increase of the basal and diastolic amplitude (Fig. 4c and d). The diastolic amplitude was only significantly higher and more prolonged at 5.5 mM. The signal detected by the isotonic transducer decreases, suggesting a more contracted heart cavity (Fig. 4d). Surprisingly, the addition of ACh at 1 mM paralysed the systolic contractions which correspond to the short amplitude pulses registered by the isotonic transducer.

### Nicotine inhibits heart function

**Fig. 5:**
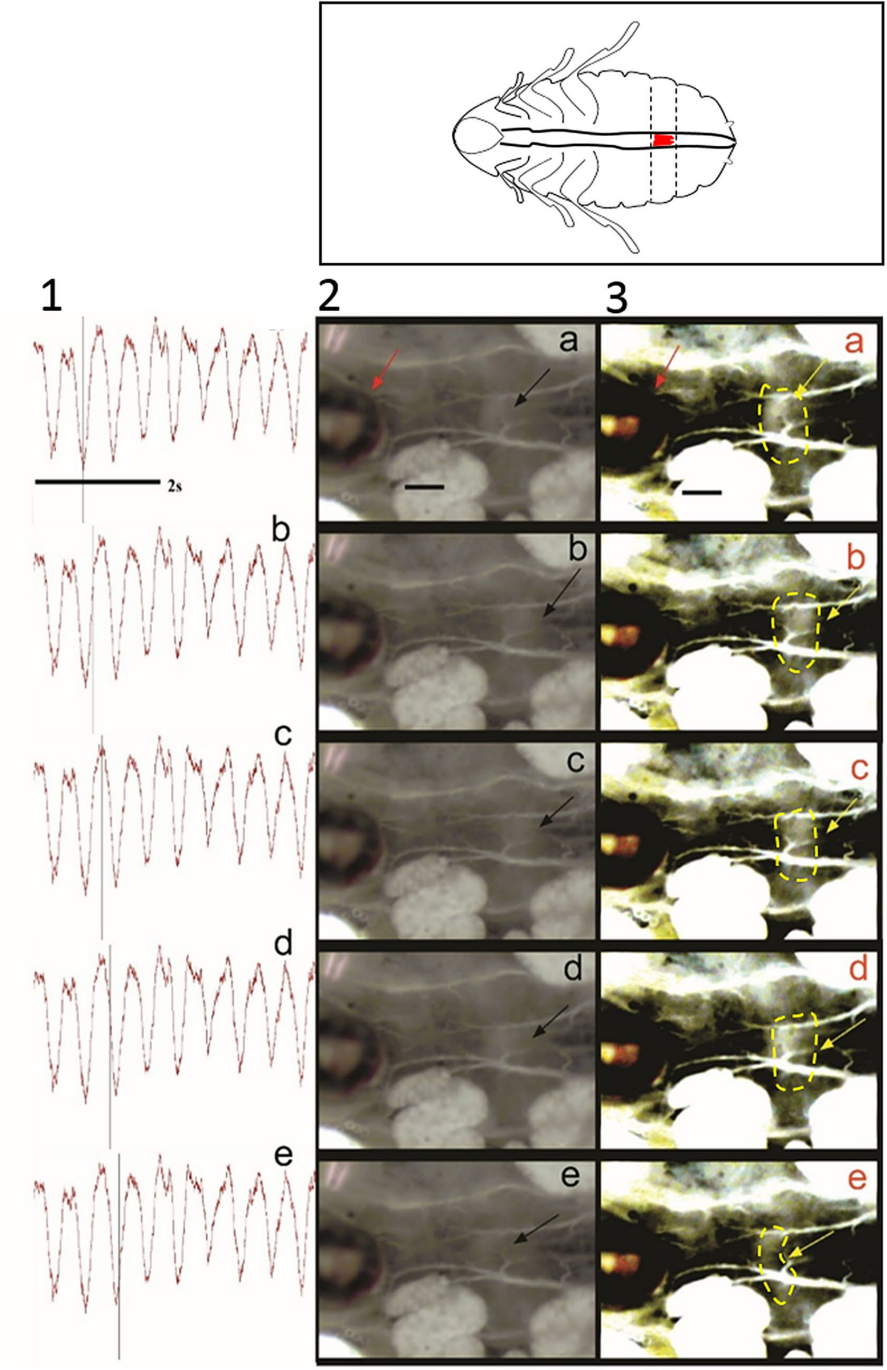
Effects of the addition of nicotine (0.53 µM and 5.3 µM) to semi-isolated heart preparations of *G. portentosa*: **a** cardiac frequency, **b** opening of heart, and **c** the diastolic amplitude of heart. The amplitudes after nicotine addition are expressed as 100% of the control amplitude. Data shown are mean values and error bars indicate SD (*n* = 6). **d** Representative traces of heart activity. The downward-facing arrows indicate the time of addition of nicotine. Differences attributed by Post Hoc TSD Tukey Test. *p* value code ‘***’ < 0.001, ‘**’ < 0.01, ‘*’ < 0.05, ‘.’ < 0.1 and ‘’ = not significant

We also characterised the cholinergic response of the heart of *G.portentosa* using nicotine as an alternative cholinergic agonist. Although both ACh and nicotine affect the same receptors, their effects on the heart were different. The normal movement of intracardiac valves was also altered by nicotine at 5.3 µM as it occurred upon ACh addition. However, nicotine effect was characterised by impairing partially the valve function and suggest that the unidirectional flow cannot be effectively sustained. Decreased function of the valve seems to case an irregular and more restricted oscillation (Online resource 3–4), although hemolymph transport across the vessel does not seem to be entirely impeded. Nicotine induced significant effects mainly at the highest concentration. A non-significant cardioaccelerator effect was observed at low concentration (0.53 µM) accompanied by a higher raw signal on the transducer (Fig. 5a and d), whereas 5.3 µM of nicotine decreased significantly the heart rate and opening diastolic. A concentration of 53.1 µM paralysed systolic contractions similarly to ACh at 1 mM. Nicotine at 0.53 µM decreased transiently the diastolic opening (Fig. 5b), whereas at 5.3 μM the diastolic opening was significantly decreased by ~ 20%. Although the diastolic opening decreased, the diastolic amplitude increased significantly at 5.3 and 0.53 μM (Fig. 5c).

### Muscarine has minor effects on the intracardiac valve

**Fig. 6:**
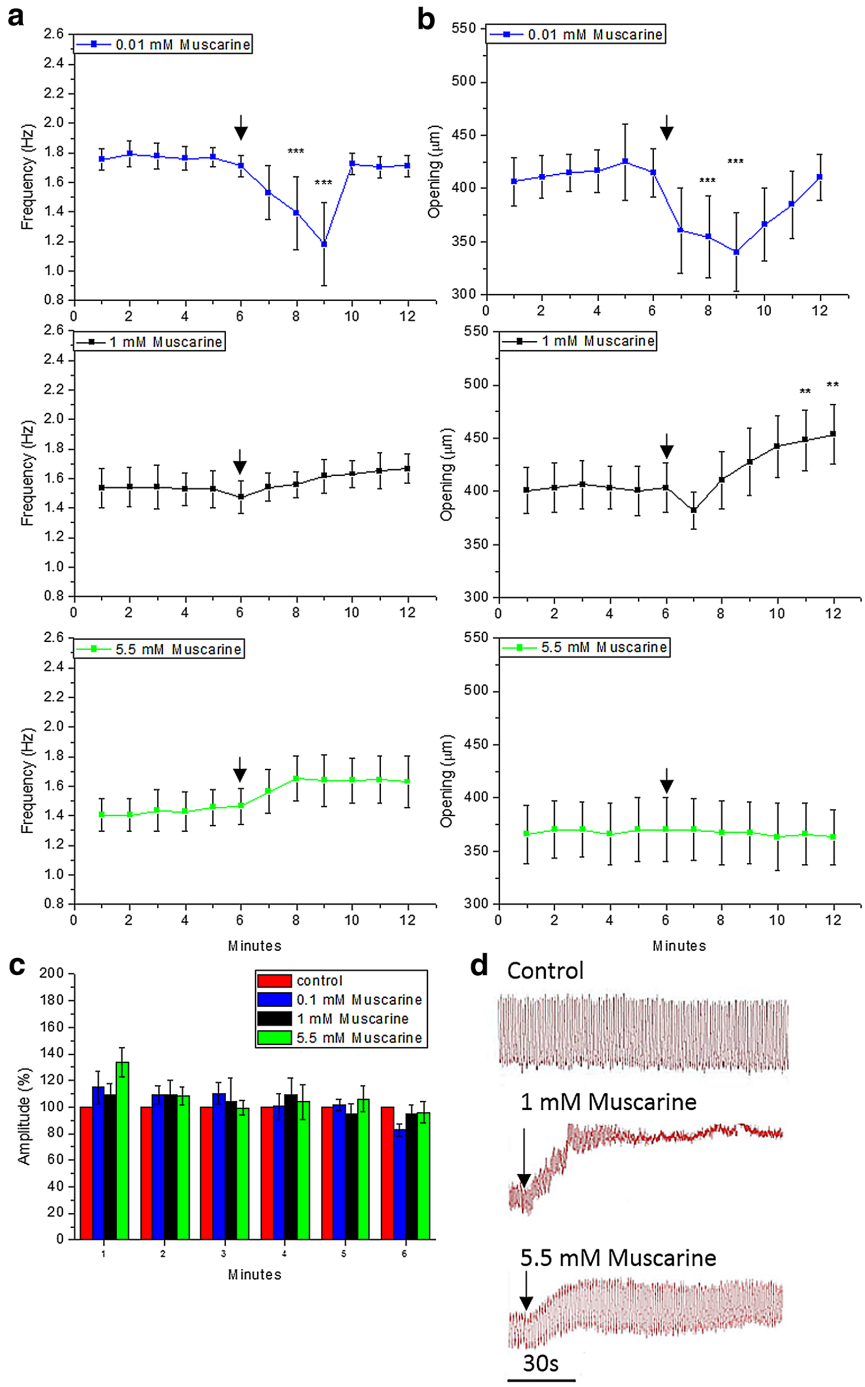
Effects of the addition of muscarine (0.01 mM, 1 mM and 5.5 mM) to semi-isolated heart preparations of *G. portentosa*: **a** cardiac frequency, **b** opening of heart, and **c** the diastolic amplitude of heart. The amplitudes after muscarine addition are expressed as 100% of the control amplitude. Data shown are mean values and error bars indicate SD (*n* = 6). **d** Representative traces of heart activity. The downward-facing arrows indicate the time of addition of muscarine. Differences attributed by Post Hoc TSD Tukey Test. *p* value code ‘***’ < 0.001, ‘**’ < 0.01, ‘*’ < 0.05, ‘.’ < 0.1 and ‘’ = not significant

As it occurred with ACh at 1 mM, muscarine at the lowest concentration (10 μM) induced a transient and significant decay of heart frequency and diastolic opening (Fig. 6a and b). In contrast, there was an increment on cardiac frequency when muscarine 1 mM and 5.5 mM were added; however, this was only found to be significant at 5.5 mM. The heart opening was increased significantly only after the addition of muscarine at 1 mM. Muscarine slightly increased diastolic amplitude (Fig. 6c). The basal amplitude increased considerably and permanently at any muscarine concentration, unlike ACh and Nicotine. Additionally, muscarine at 1 mM caused systolic paralysis similarly to ACh at 1 mM (Fig. 6d). Unlike ACh addition, muscarine did not induce any major effect on the intracardiac valves.

## Discussion

### Three-dimensional analysis of the cardiac function

The video synchronisation with the isotonic transducer recording allowed an effective study of the cardiac cycle events in a segment of the heart. Video recording of the dorsal vessel in 2 dimensions (*x, y-*axis) has been the usual approach for monitoring the heart opening of insects (Dulcis 2005; Wasserthal 2007). The addition of the signal recorded by the mechanical transducer represents a novel approach with significant advantages for monitoring the heart function such as a) the study of heart in three dimensions (*x*, *y* and *z-*axis) that provide detailed conformational changes of cardiac organs during the cardiac cycle, b) an accurate analysis the intracardiac valve relationship with each event of the cardiac cycle, and c) an easy real-time methodology for studying the effects of exogenous or endogenous agents on the heart. On the other hand, visual determination of heart section boarders can be difficult due to body fat, the thracheolus and the transparency of the heart muscle tissues. However, this limitation can be overcome by modifying the contrast of the image (Berh 2018).

### A convenient model: *Gromphadorhina portentosa*

We decided to use *G. portentosa* cockroach as a heart model because it offers many advantages over other insect models. Unlike *P. americana*, *G. portentosa* is less agile and easier to culture under captivity. It has a well-defined external and internal anatomy and a more differentiated circulatory system than *D. melanogaster* which is currently one of the most popular organisms for comparative physiology of the cardiovascular system and human cardiovascular diseases (Wasserthal 2007; Sláma 2010; Lehmacher et al. 2012). Indeed, the circulatory systems of *G. portentosa* and *D. melanogaster* share many characteristics such as 12 pairs of alary muscles and a pair of ostia between each muscle pair. The ostia have a lateral position in both species and are ended by vertical slits that form functional valves (Dailey and Graves 1976).

Based on intracardiac valve movement, the video analysis (Online resource 1) suggested that the hemolymph flow in *G. portentosa* is initiated by the diastolic phase which is longer than the systolic phase as reported on *P. americana* (Miller 1997).

Although the intracardiac valve of *G. portentosa* and *D. melanogaster* are comparable, there exist some morphological and mechanical differences which should be considered for experimental purposes. On one hand, the intracardiac valve of *G. portentosa* resemble two leaves, are very elastic and have a rotating capability of 180 degrees. In addition, the valve of *G. portentosa* oscillates back and forward following heart pulsations (Dailey and Graves 1976). On the other hand, *D. melanogaster* valves can be harder to visualise as they have round shape in the luminal space and open forming a pear shape when the luminal space is closed (Rotstein and Paululat 2016). Moreover, the semi-isolated dorsal vessel of *G. portentosa* has a larger size (3–4 inches long cockroach) and it seems to be very stable across time. Indeed, the heart rate throughout the dorsal vessel is not altered when the cockroach head is removed. In contrast, *D. melanogaster* larvae model is less stable and it loses function over time (Malloy et al. 2016). Therefore, *G. portentosa* is a more robust and reliable model for studying insect cardiac function under prolonged and severe conditions.

### The intracardiac valve is critical for the unidirectional flow of hemolymph in *G. portentosa*

The displacement of fluorescein in the open aorta and the bright field assessment of the heart contractions suggest the intracardiac valve is essential for maintaining a unidirectional/anterograde hemolymph flow and prevents any back flux as in *D. melanogaster* (Lammers et al. 2017) and in vertebrates such as the zebrafish (Armstrong and Bischoff 2004; Bettex et al. 2014). Homometabolic insects such as *D. melanogaster* and other organisms of the Endopterygota super order have periodical heartbeat reversals which consist of a direction change of peristaltic waves of the dorsal vessel (Sláma 2010; Hillyer and Pass 2020). Under physiological conditions, the video recording of the intracardiac valve sections of the dorsal vessel does not suggest conclusively the existence of these flow reversals as hemolymph is not sufficiently contrasted to determine flow directionality and the valve movement pattern does not seem to be involved in any stream direction change. Moreover, the contrasted dorsal vessel on fluorescein shows no evidence of peristaltic wave contractions driving heartbeat reversals in the heart cavity. Our findings in *G. portentosa* are in agreement with the unidirectional flow without heart reversals on *P. americana* reported by Sláma et al. (2006).

### Cholinergic responses characterisation in *G. portentosa*

The ACh effects on the isolated dorsal vessel of *G. portentosa* agree with reports on other insects (Jones 1974). The increase in cardiac frequency caused by ACh in *G. portetosa* preparations has also been documented in *D. melanogaster* (Malloy et al. 2016) and *P. americana* (Miller and Metcalf 1968).

Low ACh concentration (1 mM) in *G. portentosa* barely induced any important effect. However, the addition of a higher concentration (5.5 mM) caused irregular heartbeats, shorter diastolic periods and cardiac arrest as reported on *P. americana* (Collins and Miller 1977). Interestingly, the diameter of the heart increased during the diastolic phase increased as registered by the *x*-axis (in diastolic opening) and in *z-*axis (in diastolic amplitude). We found that not only the cavity contraction is affected, but also the movement of the intracardiac valve and possibly the net hemolymph transport. Miller and Metcalf (1968) assumed that ACh has rather a myogenic effect that propagated via the lateral cardiac nerve cord to all dorsal vessel via neuromuscular junctions and the excitation–contraction sarcolemma membranes of the myocardium. This myogenic response could also be present in *G.portentosa*.

Effects of nicotine on *G. portentosa* were only observed at higher doses and consisted of a decreased frequency of the heart which correlates with the decreased tonicity of the denervated sections of *P. americana* heart reported by Miller and Metcalf (1968). These authors suggest that the effects of nicotine are very similar to the neuronal inputs driven by ACh.

Overall, the effects of muscarine were not significant, low concentration of muscarine induced a subtle decrease of heartbeats, whereas heart frequency was slightly accelerated at high doses. These findings suggest a minor presence of functional muscarinic receptors in the cardiomyocytes of *G. portentosa*. These receptors induce a significant enhancement in pacemaker activity, leading to an increased heartbeat as observed in *D. melanogaster* (Malloy et al. 2016). The unchanged diastolic opening and amplitude upon the addition of muscarine at 5 mM suggest the presence of B-type muscarinic receptors which have constitutively low sensitivity to muscarine and are not blocked by the classical antagonists such as atropine (Collin et al. 2013).

Our experiments show that responses to cholinergic compounds in *G. portentosa* occur at higher doses compared to drosophilids. Zornik et al. (1999) reported a decrease of heart rate by ACh (1 mM–1 M) on larvae, pupae and adults of *D. melanogaster*, the nicotine increased the heart rate exclusively in adults (10 mM–100 nM), and decreased the heart rate in pupae and larvae (10 mM and 100 nM). Muscarine decreased the heart rate only in pupae (1 mM). However, these studies were performed by injecting the cholinergic agonists into the hemolymph stream of intact animals, this procedure can be particularly stressing for a relatively small and fragile organism such as the fruit fly (Malloy et al. 2016).

We observed biphasic responses of heart rhythmicity in *G. portentosa* heart, particularly after the addition of ACh and muscarin. These responses seemed to be characterised by a dose–response bell curve, such as the one reported by Hillyer et al. (2014) in *Anopheles gambiae* upon cardioactive peptides influence. Low doses of these peptides increase heart contraction rates, whereas high doses decreased heart contraction rates and alter the proportional directionality of heart contractions. More tests are needed to characterise in detail a dose–response bell curve by cholinergic agonists in *G. portentosa*.

The heart response to cholinergic agonists in *G. portentosa* seems to affect not only the valves but also the surrounding alary muscles and connective tissue as depicted in the video recordings. These muscles are supplied by neurosecretory endings which are be distributed along the vessel of *G. portentosa* (Dailey and Graves 1976). Therefore, cholinergic agonists are presumably affecting the neurogenic control of the heart in *G. portentosa*.

Although the biogenic amines and amino acids affecting heart rhythmicity are naturally different between vertebrates and insects, both groups have a myogenic contraction mechanism for their respective circulatory pumps. Thus, the nervous system modulation of the cardiovascular system of insects share some similarities with its vertebrate analogous (Hillyer 2018). It is, therefore, valid to consider *G. portentosa* cardiovascular system as a potential tool for initial tests of drugs mechanisms and treatment for diseases before testing on vertebrates.

## Conclusion

In summary, our experimental methodology allowed a simultaneous monitoring of heart and intracardiac valves function. Our results on cholinergic agonists suggest that these compounds have a concentration-dependent effect. They induced biphasic responses on cardiac frequency, cardiac cycle, and function on the intracardiac valves muscles function in *G. portentosa*. This represents a robust and more detailed approach for exploring dynamic changes in the physiology of the circulatory system in insects.

## Electronic supplementary material

Below is the link to the electronic supplementary material.

Fig. S1 (a) Dorsal vessel (dorsal view) of a moulting cockroach contrasted on fluorescein solution background. The arrows indicate the abdominal contraction and the probable flow direction. (b) Video analysis of the heart/aorta region illustrated on (a). The y-axis is the intensity with correlates to the contraction change and x-axis is the frame number. The numbers indicate the beginning of a new wave of abdominal contractions (TIF 221 kb)

Online Resource 1 Synchronisation of video and transducer recording. The recording was made on the intracardiac valve of the third abdominal segment of *G. portentosa* (MP4 6053 kb)

Online Resource 2 Video recording of the ACh effect on the intracardiac valve of the third abdominal segment of *G. portentosa* (MP4 3587 kb)

Online Resource 3 Video recording of the nicotine effect on the intracardiac valve of the third abdominal segment of *G. portentosa* (MP4 4103 kb)

Online Resource 4 Video recording of the nicotine effect on the intracardiac valve of the third abdominal segment of *G. portentosa* (MP4 5584 kb)

Online Resource 5 Video recording of the dorsal vessel (dorsal view, anterior side-image left) of a moulting cockroach contrasted on fluorescein solution background (MP4 867 kb)

Online Resource 6 Video recording of fluorescein flow in the dorsal vessel (ventral view, anterior side-image left) of a dissected *G. portentosa*. Fluorescein was applied at the anterior part of the dorsal vessel. Green fluorescence was recorded upon black light exposure (365 nm). Fluorescein entering the aorta section is indicated by orange arrows (AVI 16535 kb)

Supplementary file8 (AVI 2822 kb)
