# Supplementary figures and images for: Three-dimensional analysis of the heart function and effect cholinergic agonists in the cockroach Gromphadorhina portentosa
Source: J Comp Physiol A Neuroethol Sens Neural Behav Physiol. 2020 Sep 21;206(6):857–70. doi: 10.1007/s00359-020-01443-5 (PMC7603477; doi:10.1007/s00359-020-01443-5)

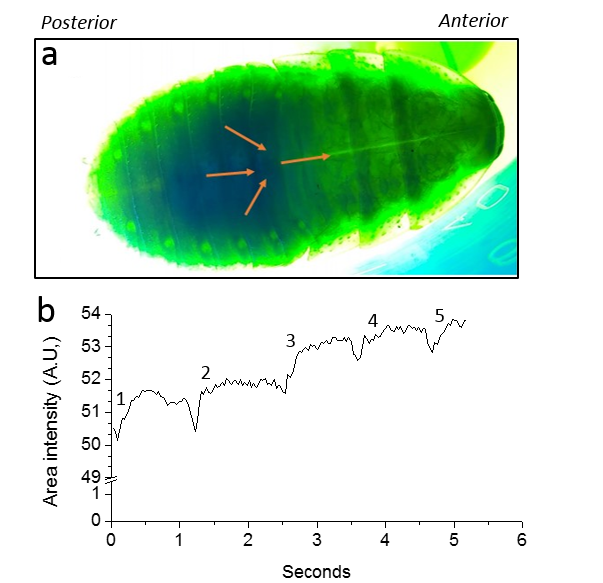

Supplement: Supplementary file 1 — Fig. S1 (a) Dorsal vessel (dorsal view) of a moulting cockroach contrasted on fluorescein solution background. The arrows indicate the abdominal contraction and the probable flow direction. (b) Video analysis of the heart/aorta region illustrated on (a). The y-axis is the intensity with correlates to the contraction change and x-axis is the frame number. The numbers indicate the beginning of a new wave of abdominal contractions (TIF 221 kb) [file 359_2020_1443_MOESM1_ESM.tif]
